# Admission Blood Glucose Level as a Predictor of Outcome in Intensive Care Patients: A Cross-Sectional Study

**DOI:** 10.7759/cureus.32801

**Published:** 2022-12-21

**Authors:** Kalaivani Subramanian, Devarajan Radha, Namitha Narayanan, Ravishankar Natarajaboopathi, Kotha Sugunakar Reddy, Divya Shanagonda, Varatharajan Sakthivadivel

**Affiliations:** 1 General Medicine, Government District Headquarters Hospital, Cuddalore, IND; 2 General Medicine, Government Villupuram Medical College, Villupuram, IND; 3 General Medicine, Stanley Medical College, Chennai, IND; 4 General Medicine, Government Thiruvannamalai Medical Collge, Thiruvannamalai, IND; 5 General Medicine, All India Institute of Medical Sciences, Hyderabad, IND; 6 General Medicine, All India Institute of Medical Sciences- Bibinagar, Hyderabad, IND

**Keywords:** roc, icu, mortality, outcome, admission blood sugar

## Abstract

Introduction: In the Intensive care unit (ICU), hyperglycemia is often observed; commonly associated with pre-existing diabetes or pre-diabetes or in nondiabetic patients. This study aimed to assess the role of admission blood sugar levels with outcomes in ICU patients.

Methods: A total of 100 patients above 18 years of age were included in the study. A detailed history regarding the patient’s age, sex, and any chronic illness were taken. Heart rate, systolic blood pressure, and Glasgow coma scale (GCS) scores were recorded. Admission blood glucose level, blood urea, total leucocyte count, and serum electrolytes were measured; and the outcome was noted.

Results: The number of diabetics was significantly higher (65.5%) in the random blood sugar (RBS) ≥180 group. Hyponatremia was significantly associated with hyperglycemia. Patients with hyperglycemia had serum bicarbonate <18. A significantly greater number of patients with hyperglycemia had GCS scores of <8, and required mechanical ventilation. The duration of ICU stay and non-survivors were significantly higher in the hyperglycemia group. Random blood sugar at admission as a factor to assess outcome showed a sensitivity of 68.4 and specificity of 59.3 with a cut-off value of 197.

Conclusion: Admission of random blood sugar was significantly associated with poor outcomes. More stringent surveillance as well as routine blood glucose checks at the time of hospital admission should be emphasized.

## Introduction

Hyperglycemia is more prevalent in intensive care units where it may not be linked to patients with diabetes or pre-diabetes. In critical care units, the prevalence of hyperglycemia increases to approximately 15% to 30% [1,2]. In addition, hyperglycemia on admission appears to be associated with an increase in long-term mortality by a factor of 1.5 and forecasts an increased risk to develop diabetes subsequently [3]. The exact cause of hyperglycemia in critical care is not fully understood, probably due to undiagnosed diabetes or pre-diabetes or due to stress induced by counter-regulator hormones and insulin resistance. Stress hyperglycemia can be defined as a blood glucose level >140mg/dl and may occur in patients with or without diabetes [1]. Although it is an adaptive response for survival, there is a strong correlation between hyperglycemia and poor prognosis in critical care [4].

Release of counter-regulatory stress hormones (cortico-steroids, catecholamines), pro-inflammatory mediators, and administration of exogenous cortico-steroids, vasopressors, and parenteral solutions containing dextrose are also possible mechanisms behind stress hyperglycemia. Apart from cortisol and epinephrine, glucagon is an important contributor to stress hyperglycemia [5]. These possible mechanisms result in excessive hepatic glycogenolysis and gluconeogenesis and insulin resistance. Further, it increases pro-inflammatory cytokine production, including IL-1, IL-6, and tumor necrosis factor (TNF)-alpha which in turn, may alter insulin receptor signaling thereby increasing insulin resistance [6].

Hyperglycemia is an adaptive response to injury or critical illness. Glucose is delivered to vital tissues such as the brain and blood cells while glucose uptake is diminished in insulin-dependent tissues such as skeletal muscle and fatty tissues. However, sustained hyperglycemia causes an increase in radical oxygen species (ROS) with subsequent mitochondrial dysfunction [7]. The mitochondrial dysfunction and change in ultrastructure are thought to be a culprit of organ dysfunction and may contribute to an increase in mortality associated with stress hyperglycemia [8]. 

Admission hyperglycemia was associated with an increase in poor outcomes and mortality in hospitalized patients presenting with an infectious disease [9]. This study aimed to address the reliability of random blood sugar (RBS) as a predictor of the outcome of critical illness.

## Materials and methods

This cross-sectional observational study was done at the tertiary care center in northern Tamilnadu after obtaining the Chengalpattu Medical College Institutional Ethics Committee (IEC) approval for one year (ECR/774/INST/TN/2015/dated 28/03/2019). A total of 100 patients were included after obtaining written informed consent. All patients above 18 years of age with admission to intensive care units were included in the study. Patients with a blood sugar level of less than 70 mgs% and those who were not willing to participate in the study were excluded from the study. A detailed history regarding the patient’s age, sex, and any chronic illness were taken. Admission blood glucose level was noted. Heart rate, systolic blood pressure, and Glasgow coma scale (GCS) scores were recorded. Blood urea, total leucocyte count, and serum electrolytes were measured. Hyperglycemic patients (RBS ≥180 mgs%) were compared with normoglycemic (RBS <180 mgs%) patients in terms of duration of stay in ICU, need for mechanical ventilation, and their outcome as survivors and non-survivors.

Statistical analysis

Microsoft Excel (Microsoft Corp., Redmond, WA, USA) was used to enter the obtained data, and SPSS (IBM Corp., Armonk, NY, USA) software version 25 was used for analysis. Mean and standard deviation were used to describe continuous data, and frequency and percentages were used to represent categorical data. To compare the categorical data between the groups, chi-square analysis was used. To forecast the outcome, the receiver-operating characteristic (ROC) was used to determine the cut-off value for RBS. Statistical significance was defined as a probability value (P) value less than 0.05.

## Results

The RBS at the time of admission of patients in the ICU is shown in Table 1. The patients were broadly divided into two groups: the group with RBS <180mg/dl and the other group with RBS ≥180mg/dl. There was no significant difference in the age group of the patients in both groups. Males were significantly greater in the RBS<180 group whereas females were greater in the RBS ≥180 group, and this distribution was significant. The presence of smokers was also comparable in both groups, while 27.8% of alcoholics were in RBS<180 groups as compared to 8.7% in the other group. The presence of co-morbidities was also almost equal; a similar distribution pattern was observed in presence of hypertension (HTN) and coronary artery disease. The number of diabetics was significantly higher (65.5%) in the RBS≥180 group.

**Table 1:** Demographic data of the study population RBS: Random blood sugar

Parameters	RBS <180 (n=54) Frequency (Percentage)	RBS ≥180 (n=46) Frequency (Percentage)	P-value
Age (Years)			0.357
< 20	-	2 (4.3)	
21 - 40	16 (29.6)	9 (19.6)	
41 - 60	18 (33.3)	25 (54.3)	
> 60	20 (37.0)	10 (21.7)	
Male	36 (66.7)	20 (43.5)	0.026
Female	18 (33.3)	26 (56.5)*	
Smoker	19 (35.2)	12 (26.1)	0.389
Alcoholism	15* (27.8)	4 (8.7)	0.021
Diabetes mellitus	9 (16.7)	30 (65.2)*	0.000
Hypertension	16 (29.6)	12 (26.1)	0.824
Coronary artery disease	4 (7.4)	8 (17.4)	0.216
Glycated Hemoglobin (HbA1C)	5.24±0.17	8.07±1.39*	0.000

Table 2 shows the clinical features and complications of the study population. The heart rate was>100 and significantly higher in the group with RBS ≥180. The systolic blood pressure, serum potassium levels and total leucocyte count did not differ significantly among the two groups. Hyponatremia was significantly associated with hyperglycemia as it can be seen that 34.8% of patients with serum sodium levels <135 had RBS ≥180. Similar results were observed for bicarbonate, patients in the RBS ≥180 group had serum bicarbonate <18. The severity when assessed by the GCS showed that a significantly greater number of patients with RBS ≥180 had GCS score of <8. Similarly, more number of patients in the group with RBS ≥180 required mechanical ventilation. The duration of ICU stays ≥5 days was significantly higher in patients with RBS ≥180. The non-survivors were significantly greater (28.7%) in the same group.

**Table 2:** Clinical features and complications of the study population RBS: Random blood sugar

Parameters	RBS <180 (n=54) Frequency (Percentage)	RBS ≥180 (n=46) Frequency (Percentage)	P-value
Heart rate			0.007
60- 100/min	46 (85.2)	28 (60.9)	
> 100/min	8 (14.8)	18 (39.1)*	
Systolic Blood Pressure (mm/hg)			0.616
< 90	8 (14.8)	10 (21.7)	
91 - 140	34 (63)	28 (60.9)	
> 140	12 (22.2)	8 (17.4)	
Blood Urea (mg/dl)			
≤40	34 (63)	28 (60.9)	0.839
> 40	20 (37)	18 (39.1)	
Total Leucocyte Count (cells/mm^3^)			0.369
< 4000	2 (3.7)	-	
400 – 11000	20 (37)	20 (43.5)	
> 11000	32 (59.3)	26 (56.5)	
Serum Sodium (mEq/L)			0.035
< 135	7 (13)	16 (34.8)*	
135 - 145	41 (75.9)	26 (56.5)	
>145	6 (11.1)	4 (8.7)	
Serum Potassium (mEq/L)			
< 3	6 (11.1)	10 (21.8)	0.313
3.1 to 4.9	44 (81.5)	34 (73.9)	
> 5	4 (7.4)	2 (4.3)	
Serum Bicarbonate (mEq/L)			0.008
< 18	6 (11.1)	10 (21.7)*	
18 - 24	40 (74.1)	20 (43.5)	
> 24	8 (14.8)	16 (34.8)	
Glasgow Coma Scale			0.033
< 8	8 (14.8)	16 (34.8)*	
9 – 15	46 (85.2)	30 (65.2)	
Mechanical Ventilation	5 (9.3)	17 (37)*	0.001
Length of Stay (days)			0.004
< 5	40 (74.1)	21 (45.7)	
≥ 5	14 (25.9)	25 (54.3)*	
Outcome			0.041
Survivors	48 (88.9)	33 (71.7)	
Non-survivors	6 (11.1)	13 (28.3)*	

**Figure 1:**
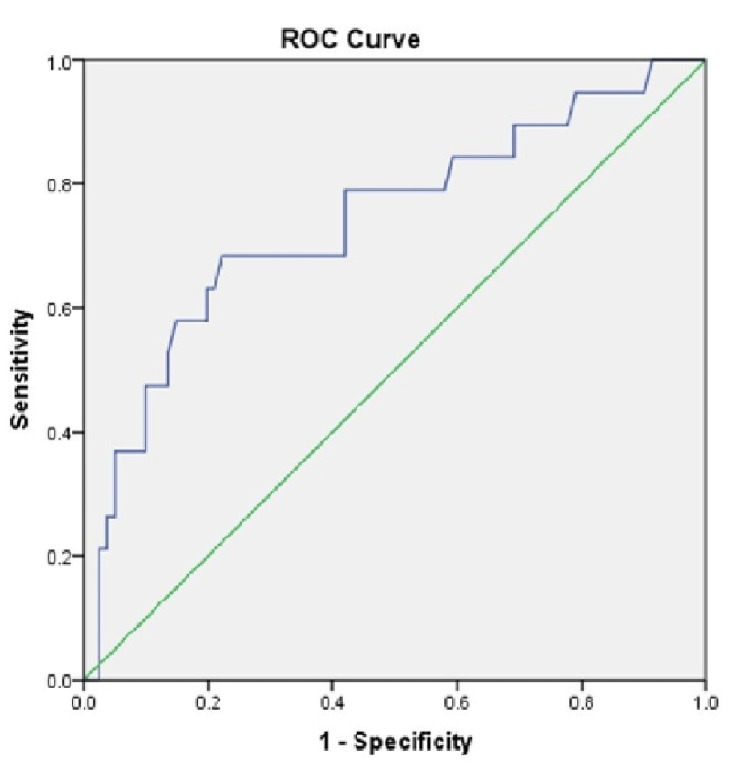
ROC curve of RBS as predictors of outcome ROC: Receiver operating characteristic curve, RBS: Random blood sugar

The performance of RBS at admission as a factor to assess outcome as assessed by the ROC curve)\ shows a sensitivity of 68.4 and specificity of 59.3 with a cut-off value of 197, and the area under the curve has a value of 0.74 (95% confidence interval (CI)-0.606-0.875) (Table 3 and Figure 1).

**Table 3:** The performance criteria of the RBS in discriminating the outcome of the study population RBS: Random blood sugar, AUC: Area under the curve, CI: Confidence interval

Parameter	AUC (95%CI)	Sensitivity	Specificity	Cut-off Value	P-value
RBS	0.74 (0.606-0.875)	68.4	59.3	197	0.001

## Discussion

In order to forecast patient outcomes and mortality with effective therapy, a variety of evaluation measures are utilized in the management of critically sick, intensive-care patients with mechanical breathing. Blood glucose levels, particularly hyperglycemia, have been extensively researched to determine how patients would respond to a variety of life-threatening circumstances, including trauma, traumatic brain injury, and burns in critical care patients. Hyperglycemia in ICU patients has been associated with increased morbidity and mortality. When increased glycogenolysis and gluconeogenesis coupled with reduced glucose uptake and reduced glycogen synthesis result in stress-induced hyperglycemia, the outcome is exacerbated in both diabetic patients and non-diabetics. Additionally, individuals with hyperglycemia are more likely to experience ischemic events, bacteremia, septicemia, and wound infections [10]. The American Diabetes Association and the American Association of Clinical Endocrinologists advise keeping blood glucose levels closer to 110 mg/dl and typically 140 mg/dl to enhance patient outcomes [11]. In the present study, most of the patients admitted to ICU were in the age group of 41 to 60 years of which 25% had RBS ≥180 mg/dl. It's noteworthy that in this study, a significant number of females than males had RBS levels above 180 mg/dl. According to Fan et al., gender substantially altered the impact of glycemic index and glycemic load on cardiovascular risk. Women were more likely to develop cardiovascular disease than males when the glycemic load was high [12]. Another study documented that there is a gender bias in the occurrence of pre-diabetic symptoms such as impaired fasting glucose (IFG) and impaired glucose tolerance (IGT). In fact, IFG is more common in males, whereas IGT is more common in women [13]. Although the cause of these discrepancies in early dysglycemia is unclear, gonadal hormones may play a role. In fact, menopausal hormone treatment lowers fasting glucose while lowering glucose tolerance. Based on the period of reproductive life, the sex difference in diabetes incidence is reversed; more diabetic males are diagnosed before puberty, whereas more diabetic women are diagnosed after menopause and as age is increased. This fact is based on the argument that, in most societies, there are greater numbers of elderly women than males as well as the fact that diabetes prevalence rises with age [14].

In the present study, a significant number of alcoholics have RBS<180mg/dl. Usha et al. found that the mean random blood sugar levels were higher in non-alcoholics than in alcoholics, but the difference was insignificant [15]. However, Leggio et al. reported high blood glucose levels in alcohol-dependent persons [16]. Alcohol-induced hypoglycemia in diabetic people is a well-known clinical issue. The mechanics behind this phenomenon, meanwhile, have mainly eluded researchers. The vagus nerve and the messenger molecule nitric oxide play key roles in how alcohol affects pancreatic microcirculation, causing a significant redistribution of blood flow from the exocrine part of the pancreas into the endocrine (which produces insulin), increasing late-phase insulin secretion, and ultimately leading to hypoglycemia [17]. Drinking alcohol in moderation may reduce the chances of developing diabetes and metabolic syndrome. However, heavy drinking has also been related to higher glucose levels, which increases the risk of metabolic syndrome and diabetes [18]. Another study found a relationship between frequent heavy drinking and binge drinking in women after the age of 16, which is a key cause of type 2 diabetes at a later age [19].

In the present study, the number of patients with co-morbidities did not vary much among the two groups except for diabetes. Previous studies examining the relationship between the existence of co-morbidities and glycemic control have shown inconsistent results. Studies have indicated no link or an inverse relationship between co-morbidities and HbA1c when the overall number of co-morbidities was analyzed [20]. While no significant connection with chronic kidney disease (CKD) was discovered in a study looking at particular co-morbidities, participants with HTN and dyslipidemia had reduced odds of attaining their glycemic goal [21]. Another study stated that having diabetes and CKD or obesity increased the likelihood of developing poor glycemic control and elevated blood glucose level, in contrast to not having these concomitant conditions. Hypertension, however, was shown to have no connection with elevated blood glucose levels [22]. Similarly, a link between obesity and inadequate glycemic control was discovered in a cross-sectional study of the Iranian population with diabetes [23]. A high incidence of inadequate glycemic management might harm healthcare systems. A cross-sectional study in Spain showed that people with poor glycemic control, elevated blood sugar, and associated co-morbidities had higher overall health-related expenses, including hospitalization and prescription costs, compared to those with good glycemic control [24].

In the present study, a significant number of patients with RBS ≥180 mg/dl had heart rates greater than 100. In a study done by Valensi et al., it was shown that patients with diabetes had a lesser increase in heart rate [25]. The incidence of type 2 diabetes was found to be correlated with heart rate in a study of the Chinese population; specifically, those with heart rates greater than 86 beats per minute. They also noted a positive dose relationship between heart rate and elevated blood sugar (P < .001) [26]. By lengthening the QT interval, the hyperglycemic condition can directly affect the electrical status of the heart, raising the risk of ventricular tachycardia (VT) and other significant cardiac arrhythmias. Hoang et al. discovered that myocardial infarction patients with blood glucose levels of more than 140 mg/dl had an increased chance of VT significantly [27]. The increased risk of VT in AMI patients with elevated glucose levels emphasizes the need for more stringent surveillance as well as routine blood glucose checks at the time of hospital admission.

In the present study, a significant number of patients with RBS ≥180 mg/dl had low sodium as well as low bicarbonate. In research by Khan et al., a substantial decrease in serum sodium and chloride levels and a rise in serum potassium levels were seen in association with rising fasting blood glucose [28 ]. Parmar et al., in their study too, reported that serum sodium and chloride levels decreased with increasing blood sugar levels. One well-known physiological source of dysnatremia in diabetes is osmotic diuresis. In people with uncontrolled diabetes, blood sodium levels fluctuate depending on how water is moved out of cells by hyperglycemia, which decreases sodium; and glucosuria, which increases sodium. Hyponatremia results from elevated blood glucose levels pulling water out of cells and into extracellular spaces [29].

In the present study, a significant number of patients had a poor GCS score of <8 and required mechanical ventilation, a greater length of stay and had a poor outcome among the group with RBS ≥180mg/dl. In their study, Terzioglu et al. discovered that patients with mean glucose values (MGV) between 150 and 179 had hazard rates that were 3.691 times higher than those of patients with MGV values between 110 and 149. Patients with MGV levels above 180 had a hazard rate that was 6.571 times higher than patients with MGV values between 110 and 149. They also concluded that in ICU patients receiving mechanical ventilation who have had traumatic brain injuries, high glucose levels are an independent risk factor for death [30]. Griesdale et al. also found a link between elevated glucose levels and a higher death rate in traumatic brain injury patients [31]. In their study of the connection between hyperglycemia at admission and neurological outcome, Young et al. found that the GCS score was low with an admission sugar level of more than 200 mg/dl [32]. Mahmoodpoor et al. found a significant relationship between blood sugar level and mortality with a relative risk of 1.01 [33].

In our study, RBS showed a sensitivity of 68.4%, specificity of 58.3, and AUC of 0.74 with a cut-off value of 197 mg/dl in predicting the outcome. A Nice sugar study stated that the outcome was better with a blood sugar level of 180 mg/dl in ICU patients [34]. In Rau et al.'s study, a blood sugar of 233 mg/dl had an AUC of 0.62 to predict the outcome in hospitalized patients with trauma [35].

Limitations

This is a single-center study with a sample size of hundred patients. However, our study confirms the poor prognosis of blood sugar levels in intensive care patients. Large-scale multicenter studies involving various ethnic population is needed to homologate these findings.

## Conclusions

Hyperglycemia at the time of admission is very common in intensive care units. Admission blood sugar level was associated with poor outcomes in intensive care patients. These patients are more prone to various complications like infections, ischemic events, and mortality.

In our study, admission blood sugar level was higher in females and patients with diabetes mellitus. A significant number of patients with hyperglycemia had a GCS score of <8 and required mechanical ventilation. The duration of ICU stay and non-survivors were significantly higher in the hyperglycemia group. Hence, more stringent surveillance as well as routine blood glucose checks at the time of hospital admission should be emphasized.
